# Supplementary material for: Respiratory Syncytial Virus Infection Induces Chromatin Remodeling to Activate Growth Factor and Extracellular Matrix Secretion Pathways
Source: Viruses. 2020 Jul 26;12(8):804. doi: 10.3390/v12080804 (PMC7472097; doi:10.3390/v12080804)

Supplemental Table S1. Mapped reads for ATAC-Seq dataset

Dataset	No. Reads	No. Mapped	Percentage (%)
**WT_1**	46,036,987	40,360,835	88
**WT_2**	59,175,288	51,987,516	88
**RSV_1**	58,691,358	51,326,986	87
**RSV_2**	56,720,822	49,220,216	87

Supplemental Figure S1. Quality assessment of ATAC-Seq data. For each replicate, Fragment size and library complexity is plotted.

S1A. Fragment size, control sample, replicate 1

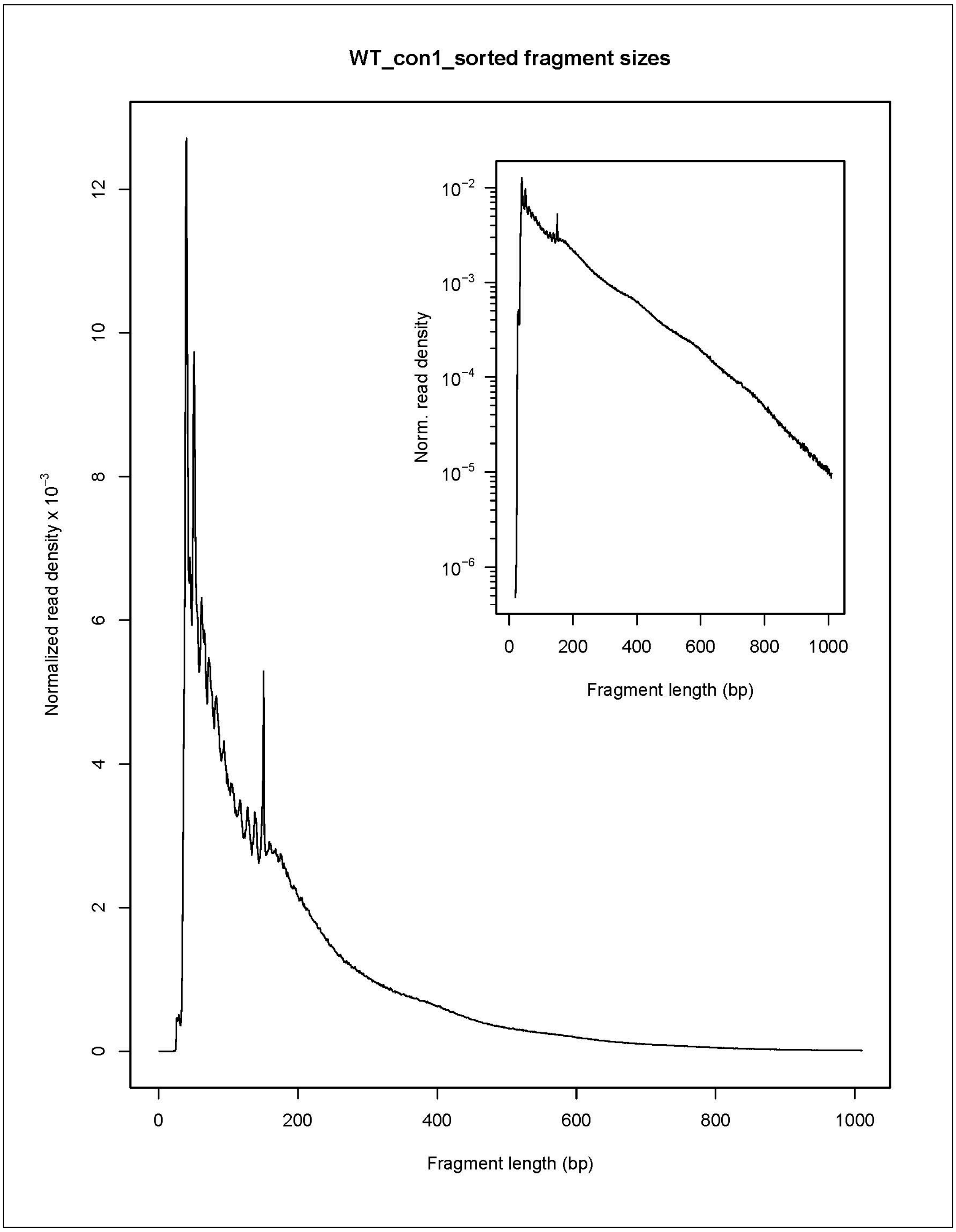

S1B. Library complexity, control sample, replicate 1

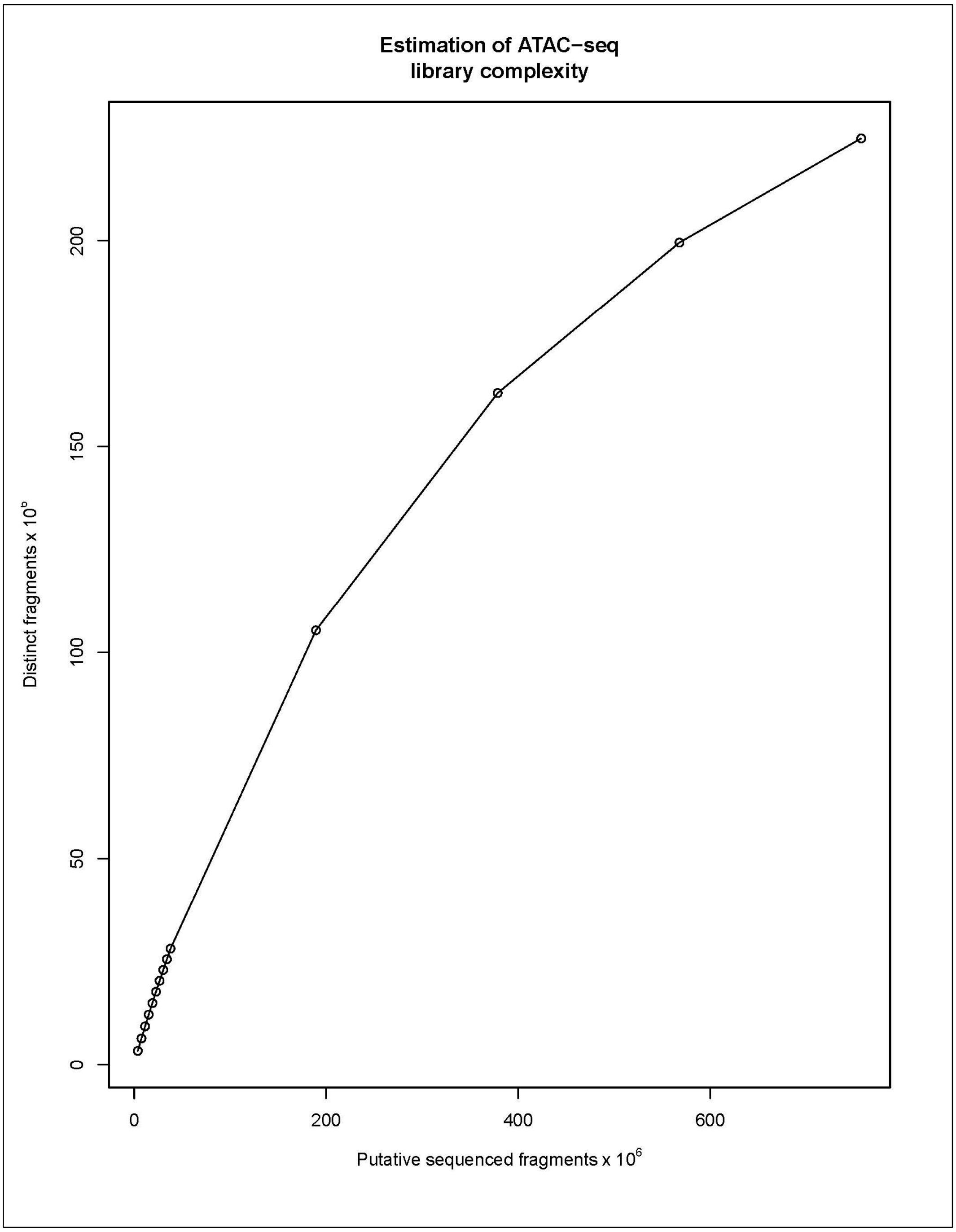

S1C. Fragment size, control sample, replicate 2

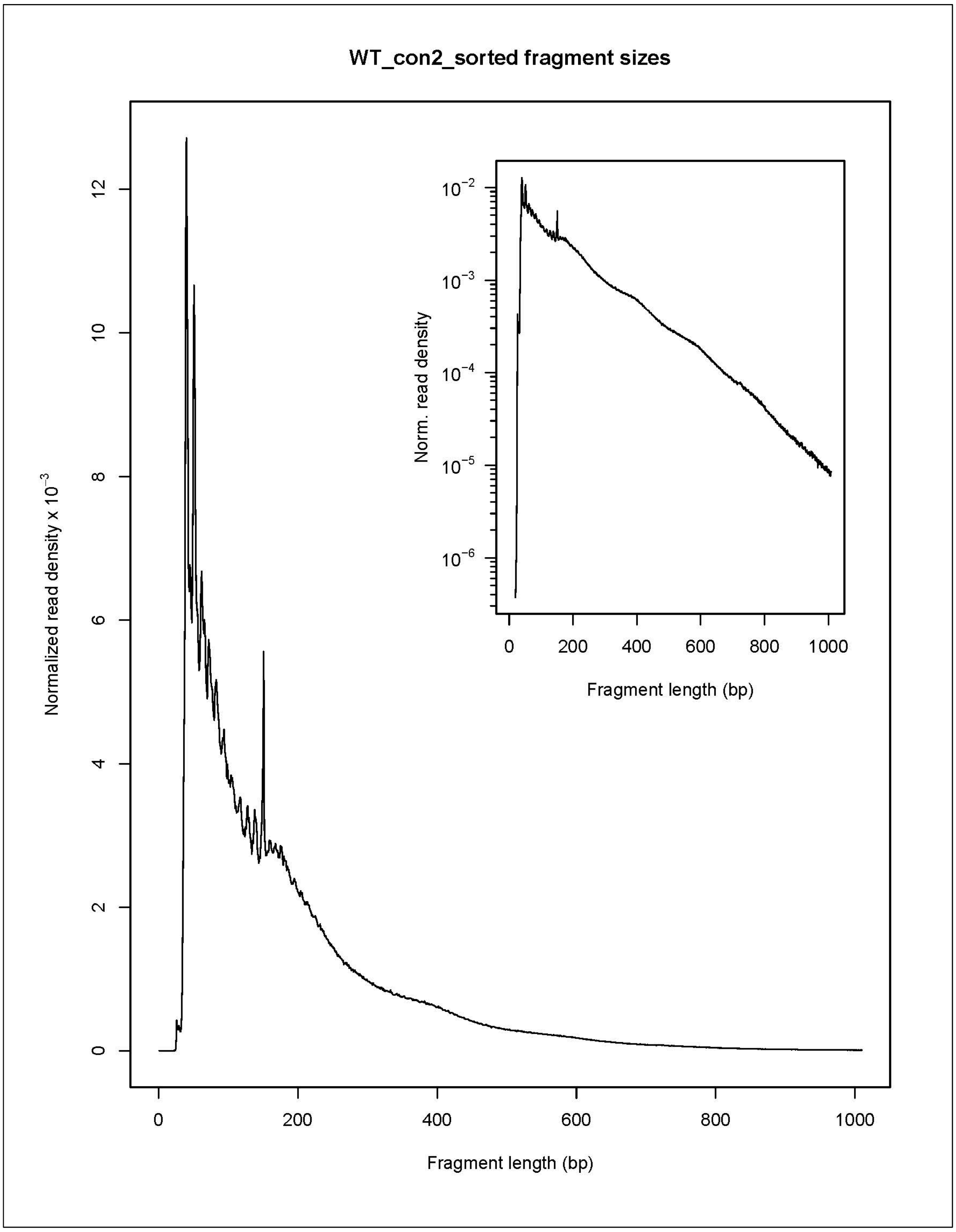

S1D. Library complexity, control sample, replicate 2

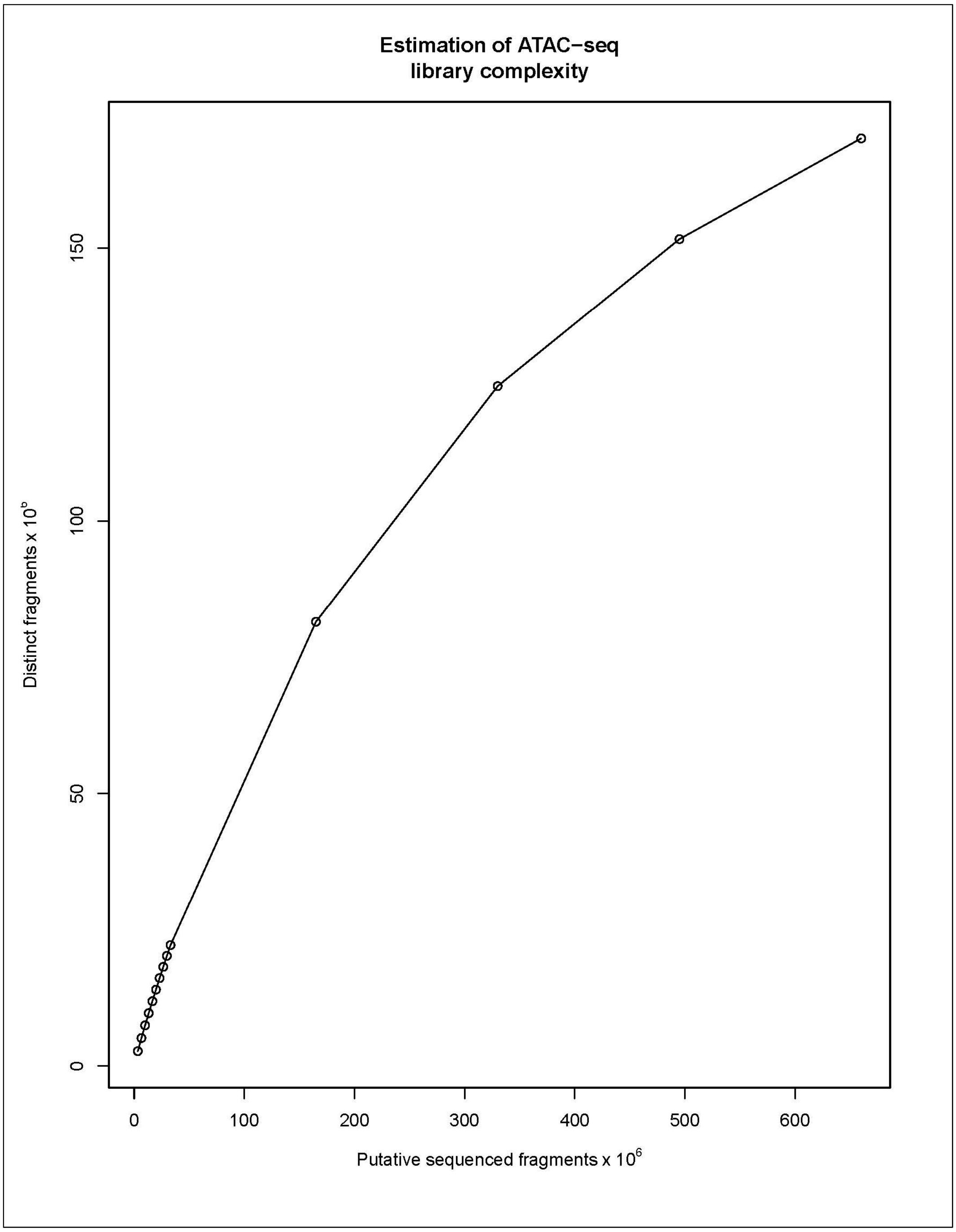

S1E. Fragment size, RSV infected, replicate 1

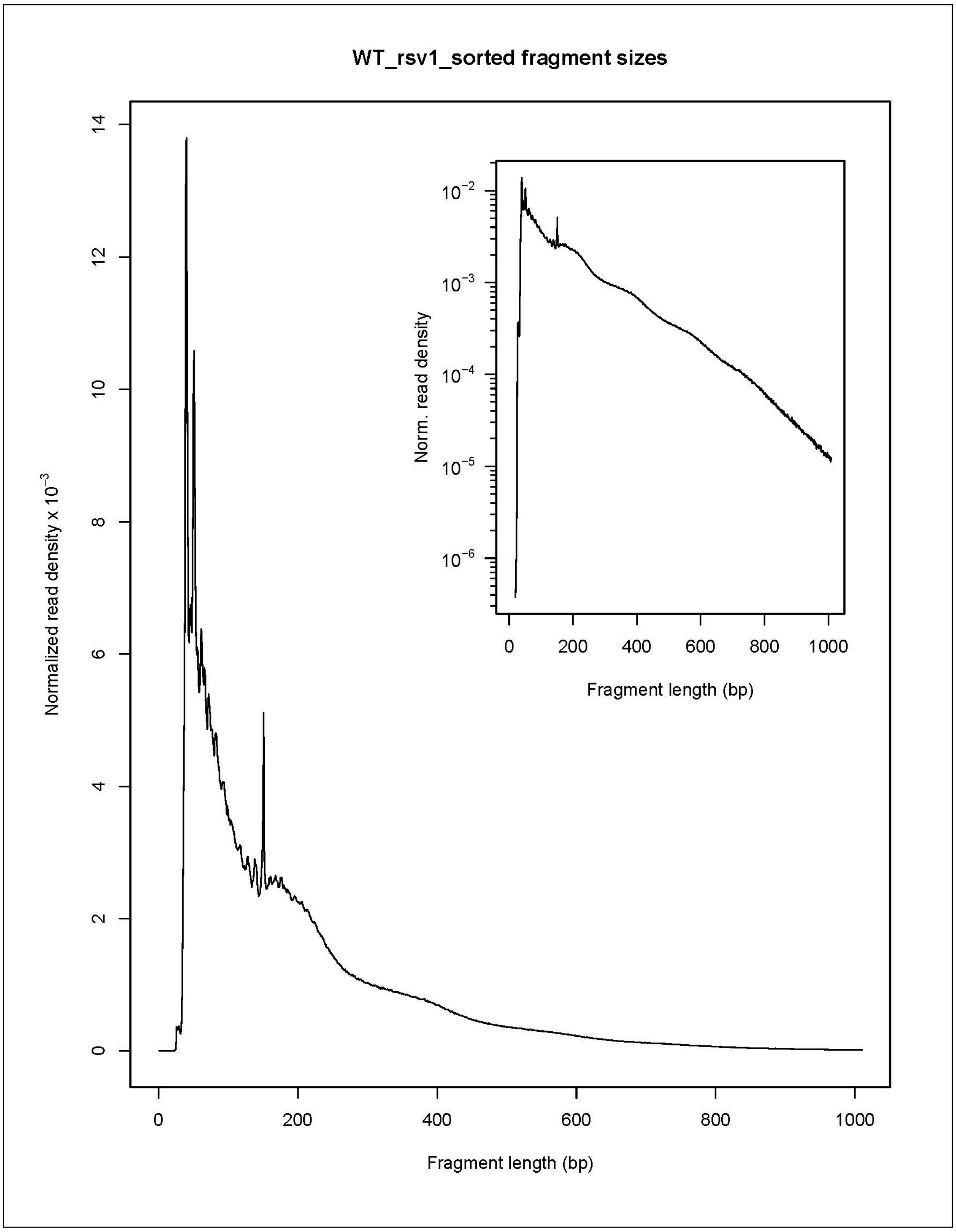

S1F. Library complexity, RSV sample, replicate 1

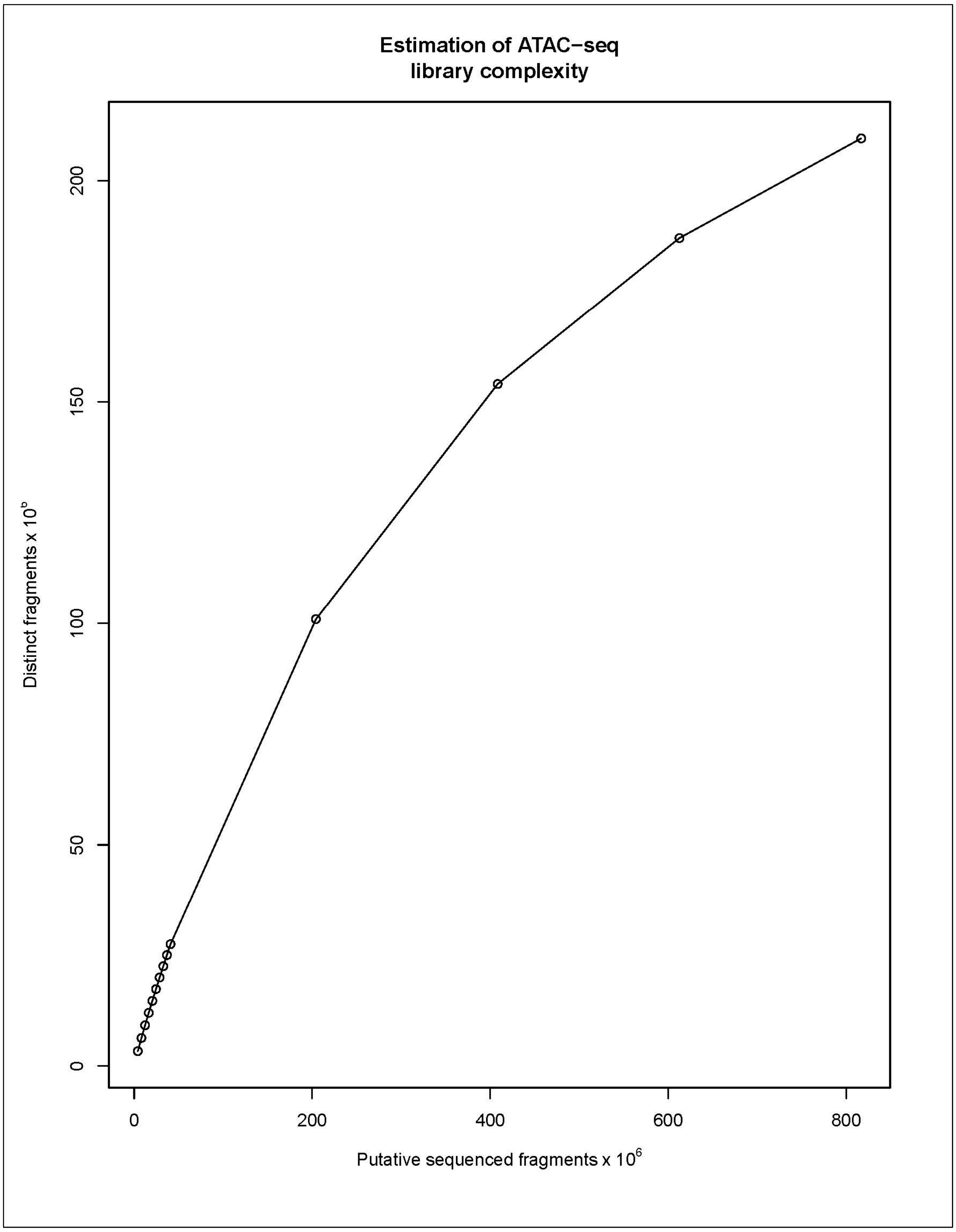

S1G. Fragment size, RSV sample, replicate 2

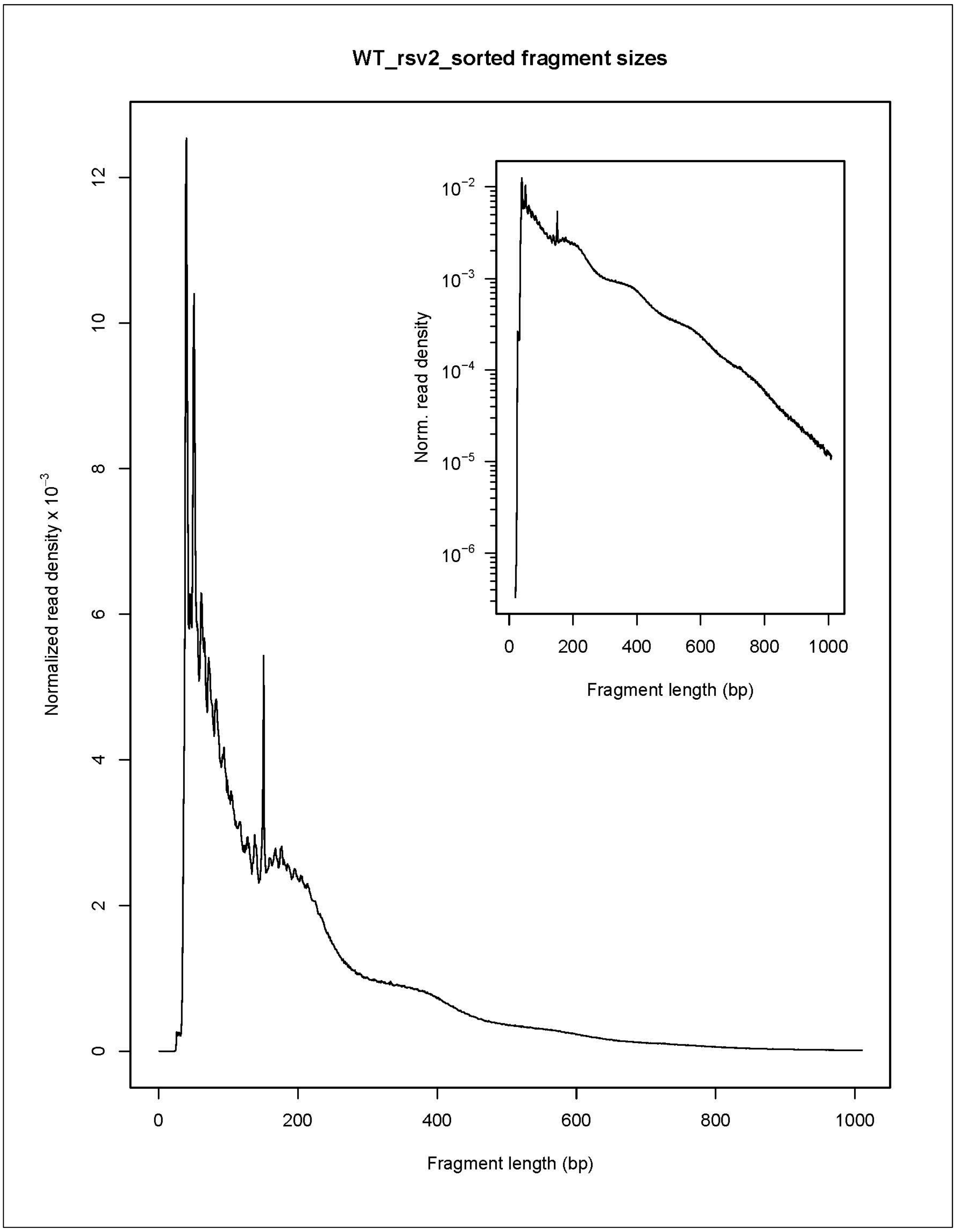

S1H. Library complexity, RSV sample, replicate 2

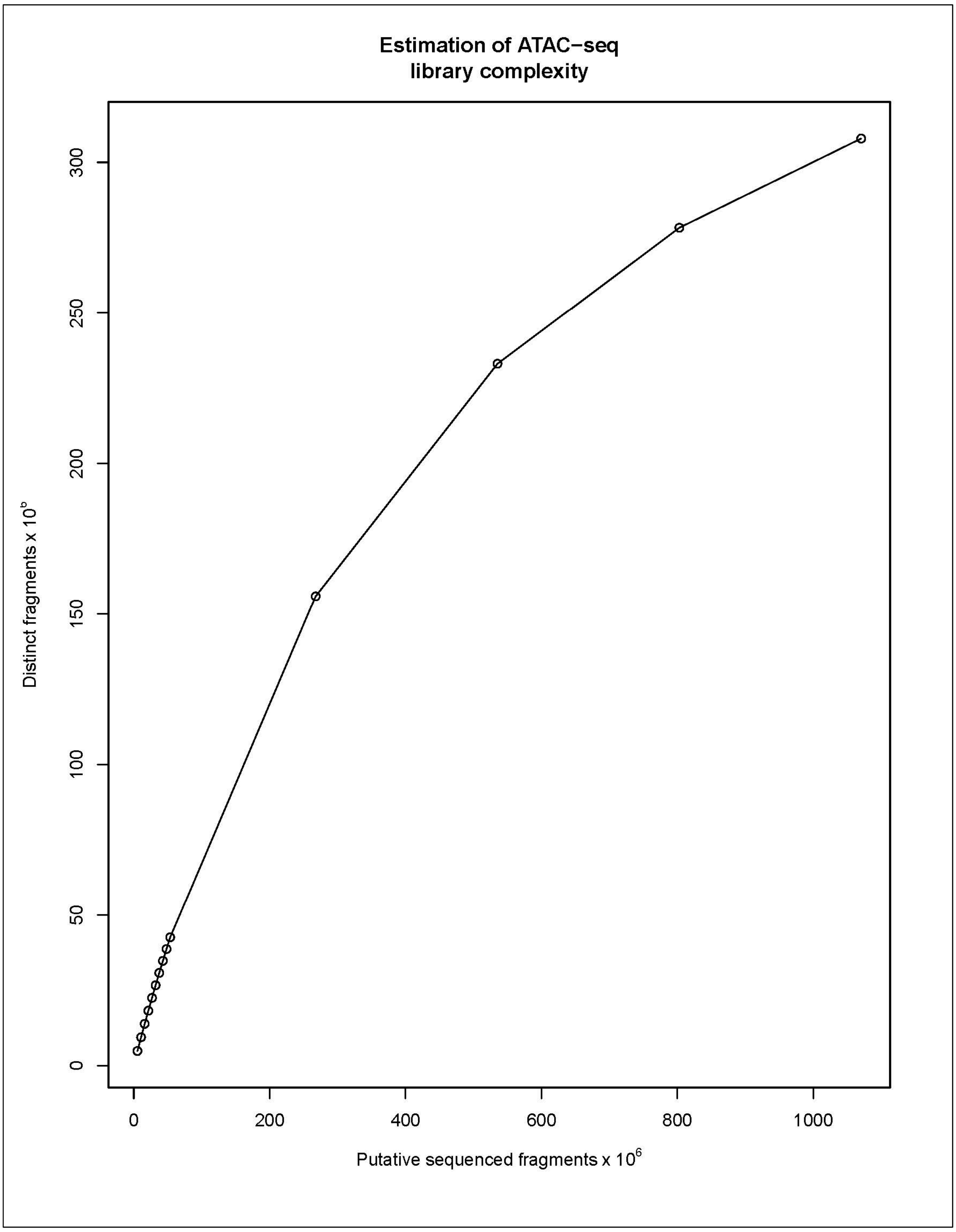

Supplemental Figure 2. Overlap of read calling algorithms for occupancy analysis. Shown are number of unique genes with ATAC-Seq peaks ± 3 kb of TSS identified in uninfected hSAECs.

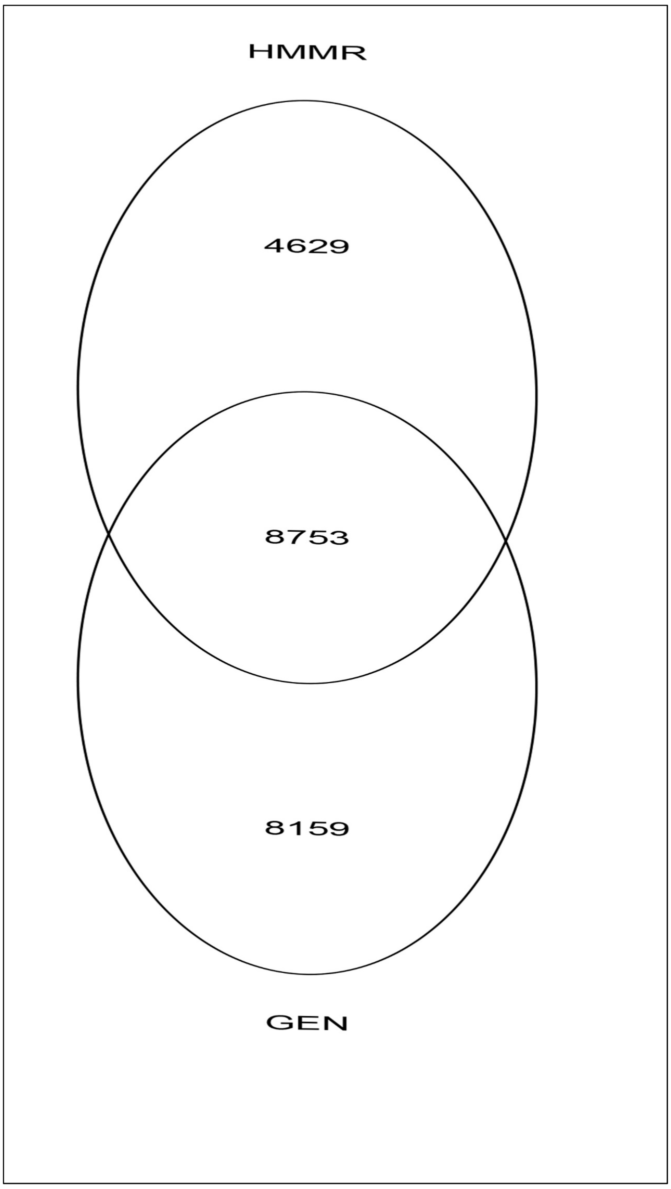

Supplemental Figure 3. Overlap of read calling algorithms for differential analysis. Shown are number of unique genes with ATAC-Seq peaks ± 3 kb of TSS identified in RSV-infected vs uninfected hSAECs.

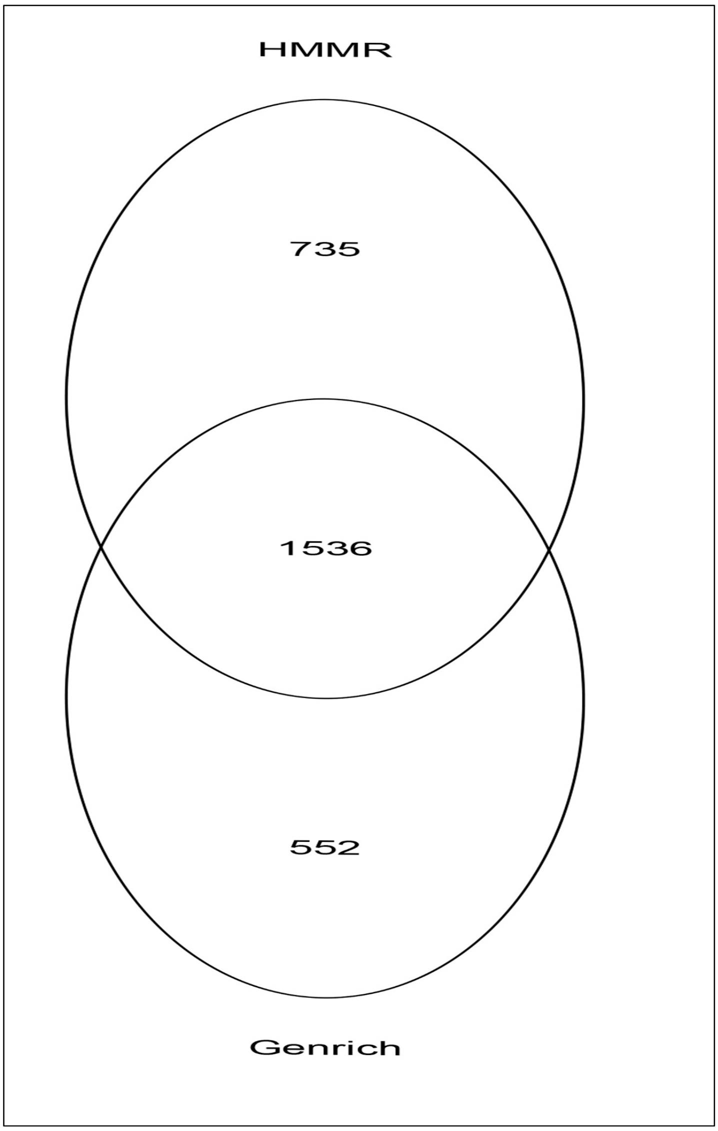

Supplement: Supplementary file 1 [file viruses-12-00804-s001.pdf]
